# 

**Published:** 1869-05-15

**Authors:** 


### CHICAGO

### Medical Journal.

PUBLISHED SEMI-MONTHLY.

J. ADAMS ALLEN, M. D., LL. D.,

*EDITOR.*

WALTER HAY, A. M., M. D.,

*ASSOCIATE EDITOR.*

MAY 15, 1869.

CHICAGO:

HORTON & LEONARD, STEAM BOOK AND JOB PRINTERS,

104 & 106 Randolph Street.

